# In This Issue

**Published:** 1998

## THE ENDOCRINE SYSTEM: AN OVERVIEW

The endocrine system plays a leading role in a variety of bodily functions, including growth, development, metabolism, and reproduction. This article reviews the complex mechanisms that are at the heart of the body’s endocrine system, including the hypothalamus, the chief orchestrator of the endocrine system; the pituitary, the master gland of the body, which is responsible for producing and releasing a wide variety of hormones; and the various hormones themselves, the chemical signals that are involved in endocrine-system communication. Drs. Susanne Hiller-Sturmhöfel and Andrzej Bartke review those brain regions and glands, the major hormones they produce, and the functions of those hormones. (pp. 153–164)

## ALCOHOL AND FEMALE PUBERTAL DEVELOPMENT

Girls who consume alcohol during early adolescence place themselves at increased risk for delayed puberty and other hormonal consequences. Drs. W. Les Dees, Jill K. Hiney, and Vinod Srivastava describe research linking those disturbances to alcohol’s effects on an essential hormone, insulin-like growth factor 1 (IGF-1). Produced by the liver, IGF-1 acts within the brain to help coordinate physical growth with the maturation of the reproductive system. Results of animal studies suggest that long-term alcohol consumption may suppress production of IGF-1 by the liver, whereas episodes of binge drinking can block IGF-1 function within the brain. If those effects occur in humans, they may inhibit the release of essential reproductive hormones required to initiate female puberty. (pp. 165–169)

## HORMONAL EFFECTS OF ALCOHOL USE ON THE MOTHER AND FETUS

The mother’s hormone system undergoes extensive changes to sustain the developing fetus. At the same time, the fetus gradually develops its own hormone system. Maternal alcohol consumption during pregnancy can interfere both with the mother’s hormone activities and—because alcohol readily crosses the placenta—with the developing fetal hormone system. Ms. Kara Gabriel, Ms. Candace Hofmann, Ms. Maria Glavas, and Dr. Joanne Weinberg review alcohol’s numerous effects on hormone functioning in both the mother and the fetus. Alcohol-induced changes in hormone activities may be responsible for some of the characteristics (e.g., hyperactivity and impulsive behavior) commonly observed in children who were prenatally exposed to alcohol. (pp. 170–177)

## ALCOHOL AND THE HORMONAL CONTROL OF LACTATION

In mammals, including humans, milk production (i.e., lactation) is an essential component of reproduction, ensuring nourishment of the offspring. Several hormones regulate both the initiation and maintenance of lactation, report Drs. Sarah H. Heil and Marappa G. Subramanian. The most important of those hormones are prolactin and oxytocin. The production of those hormones is stimulated by changes in the mother’s hormonal balance as well as by the offspring’s suckling. Maternal alcohol consumption may inhibit the release of both prolactin and oxytocin, thereby interfering with effective lactation. In addition, maternal alcohol consumption during lactation can alter the milk’s composition and may impair the offspring’s suckling behavior. Animal studies also indicate that maternal alcohol exposure even long before pregnancy may affect lactation by interfering with normal mammary gland development. (pp. 178–184)

## ALCOHOL AND POSTMENOPAUSAL WOMEN

Hormone replacement therapy (HRT) is commonly prescribed to ameliorate symptoms of menopause. The use of HRT produces mixed health effects and is therefore controversial. Studies show that moderate alcohol consumption may increase estrogen levels in postmenopausal women receiving HRT. Such alterations in hormonal balance may affect the risk for osteoporosis, heart disease, and cancer. Drs. Matthew P. Longnecker and Marilyn Tseng review the hormonal changes associated with menopause, examine the effects of alcohol consumption on postmenopausal levels of estrogens and related hormones, and discuss potential positive and negative health consequences of alcohol use after menopause. (pp. 185–189)

## ALCOHOL’S HARMFUL EFFECTS ON BONE

As a living tissue, bone is susceptible to the adverse effects of alcohol consumption. Drinking during adolescence increases the risk of bone disease later in life. Adult drinking weakens the skeleton by interfering with the remodeling of bone that continues throughout life. Dr. H. Wayne Sampson summarizes alcohol’s harmful effects on bone and suggests possible physiological mechanisms for their development. Alcohol exerts effects both directly and indirectly through the many cell types, hormones, and growth factors that regulate bone metabolism. Alcohol appears to weaken bone largely by inhibiting the function of specialized cells that deposit new layers of bone during remodeling. (pp. 190–194)

## ALCOHOL’S EFFECTS ON MALE REPRODUCTION

The male reproductive system consists of the hypothalamus, which produces gonadotropin-releasing hormone (GnRH); the pituitary gland, which produces both luteinizing hormone (LH) and follicle-stimulating hormone (FSH); and the testes, which produce testosterone. Alcohol can interfere with the function of each of those organs and hormones, report Drs. Mary Ann Emanuele and Nicholas V. Emanuele. In the testes, alcohol can lead to tissue shrinkage, reduced testosterone production, and sperm abnormalities. These alcohol-induced hormonal changes may contribute to male infertility and to various other deleterious effects throughout the body, such as bone loss and decreased muscle function. (pp. 195–201)

## ALCOHOL-SEEKING BEHAVIOR

The body’s response to stressful situations involves two important hormone systems—the hypothalamic-pituitary-adrenal (HPA) axis and the endogenous opioid system. Alcohol consumption, like stress, can activate both of these hormone systems, leading to the production of key stress hormones. Dr. Christina Gianoulakis describes how those stress hormones act on various brain regions to induce alcohol’s pleasurable effects. She also describes how stress hormones may be involved in alcohol craving. Genetic differences in the body’s hormonal response to stress and alcohol may determine a person’s sensitivity to alcohol’s pleasurable effects and his or her susceptibility to developing alcoholism. (pp. 202–210)

## CONSEQUENCES OF ALCOHOL USE IN DIABETICS

In people with diabetes, blood sugar regulation by the hormone insulin as well as fat and protein metabolism are impaired, either because the pancreas produces insufficient insulin or because the body is unresponsive to insulin’s actions. Those metabolic disturbances can result in serious medical problems, such as cardiovascular, neurological, and eye complications. Drs. Nicholas V. Emanuele, Terrence F. Swade, and Mary Ann Emanuele report that regular alcohol consumption, even of moderate amounts, can interfere with blood sugar control in diabetics. Particularly when combined with inadequate food intake, alcohol can induce potentially life-threatening declines in blood sugar levels in diabetics. In addition, alcohol consumption can increase the risk of diabetes-associated medical complications. (pp. 211–219)

## ALCOHOLIC BEVERAGES AS A SOURCE OF ESTROGENS

The effects of alcoholic beverages on the body may result not only from the alcohol itself but also from other substances (i.e., congeners) present in those beverages. Dr. Judith S. Gavaler explores the hypothesis that those congeners can exert estrogenlike effects that may be responsible for some of the adverse effects of excessive drinking, such as the enlarged breasts and redistributed body fat observed in some male alcoholics. Dr. Gavaler reviews studies using subjects who no longer produced estrogens themselves (i.e., female rats whose ovaries had been removed and postmenopausal women). In those studies, the administration of various alcoholic beverage congeners resulted in hormonal changes consistent with the presence of substances with estrogenlike activities in the congeners. (pp. 220–227)

